# Microstructural Abnormalities of White Matter Across Tourette Syndrome: A Voxel-Based Meta-Analysis of Fractional Anisotropy

**DOI:** 10.3389/fneur.2021.659250

**Published:** 2021-09-09

**Authors:** Chengmin Yang, Li Yao, Naici Liu, Wenjing Zhang, Bo Tao, Hengyi Cao, Qiyong Gong, Su Lui

**Affiliations:** ^1^Department of Radiology, Huaxi MR Research Center, West China Hospital of Sichuan University, Chengdu, China; ^2^Psychoradiology Research Unit of Chinese Academy of Medical Sciences, Functional and Molecular Imaging Key Laboratory of Sichuan Province, West China Hospital of Sichuan University, Chengdu, China; ^3^Center for Psychiatric Neuroscience, Feinstein Institute for Medical Research, Manhasset, NY, United States; ^4^Division of Psychiatry Research, Zucker Hillside Hospital, Glen Oaks, NY, United States

**Keywords:** Tourette syndrome, diffusion tensor imaging, fractional anisotropy, magnetic resonance imaging, meta-analysis

## Abstract

**Introduction:** Tourette syndrome (TS) is a neuropsychiatric disorder with multiple motor and vocal tics whose neural basis remains unclear. Diffusion tensor imaging (DTI) studies have demonstrated white matter microstructural alternations in TS, but the findings are inconclusive. In this study, we aimed to elucidate the most consistent white matter deficits in patients with TS.

**Method:** By systematically searching online databases up to December 2020 for all DTI studies comparing fractional anisotropy (FA) between patients with TS and healthy controls (HCs), we conducted anisotropic effect size-signed differential mapping (AES-SDM) meta-analysis to investigate FA differences in TS, as well as performed meta-regression analysis to explore the effects of demographics and clinical characteristics on white matter abnormalities among TS.

**Results:** A total of eight datasets including 168 patients with TS and 163 HCs were identified. We found that TS patients showed robustly decreased FA in the corpus callosum (CC) and right inferior longitudinal fasciculus (ILF) compared with HCs. These two regions preserved significance in the sensitivity analysis. No regions of increased FA were reported. Meta-regression analysis revealed that age, sex, tic severity, or illness duration of patients with TS were not linearly correlated with decreased FA.

**Conclusion:** Patients with TS display deficits of white matter microstructure in the CC and right ILF known to be important for interhemispheric connections as well as long association fiber bundles within one hemisphere. Because the results reported in the primary literature were highly variable, future investigations with large samples would be required to support the identified white matter changes in TS.

## Introduction

Tourette syndrome (TS) is a common neuropsychiatric disorder characterized by rigid, repetitive, and intermittent movements and vocalizations termed tics (1). The prevalence of TS is 0.3–1% in school-aged children (2, 3). TS patients with behavioral comorbidities [e.g., attention deficit hyperactivity disorder (ADHD) or/and obsessive compulsive disorder (OCD)] are associated with impaired social function and reduced overall life quality (2). A large number of neuroimaging studies have detected brain functional and structural abnormalities of TS especially involving cortico-basal ganglia-thalamo-cortical network (4–8), which were linked to the emergence of tics (6). In addition, widespread dysconnectivity involving parietal, temporal, and occipital lobes and interhemispheric brain abnormalities have also been demonstrated in TS, which may relate to mental and behavioral changes in TS patients (9–16). Since normal structure of white matter tracts is critical for maintaining connections between distant brain regions, studying white matter alterations is crucial to elucidate potential neurobiological mechanisms underlying TS. In addition, it may also highlight potential neuroimaging markers related to tic severity and neurobehavioral abnormalities in TS patients (17).

Diffusion tensor imaging (DTI) is a particularly useful technique for investigating white matter abnormalities (18), while fractional anisotropy (FA) is widely used to measure the degree of directionality of cellular structures within the fiber tracts which reflect microstructural integrity of the brain (19–21). The voxel-based analysis (VBA) and tract-based spatial statistics (TBSS) method are commonly used to investigate whole-brain FA differences. The VBA technique measures contiguous clusters of significant white matter voxels with the correction of multiple comparisons and noise. The TBSS calculates significant clusters within white matter skeletons after isolating the central core of white matter tracts with the highest FA (22). Further meta-analysis has successfully combined both methods in one study (23). Widespread white matter abnormalities of TS have been revealed by DTI studies, but the results have been inconclusive. Specifically, while some studies revealed decreased FA in corpus callosum (CC) (11, 24), bilateral superior longitudinal fascicle (11), bilateral frontal lobe (11, 25), and left external or internal capsule region (11, 24), others identified increased FA in the left postcentral gyrus (26) or no significant FA differences between TS patients and healthy controls (HCs) (17, 27–29). This inconsistency might be due to sample size, sample heterogeneity, and/or methodological differences. Therefore, a whole-brain meta-analysis identifying reliable neurobiological markers of TS is of particular importance.

In this study, we performed a meta-analysis to determine white matter abnormalities in patients with TS *via* anisotropic effect size-signed differential mapping (AES-SDM) (30), which is an effective meta-analytic technique to quantify reproducibility of neuroimaging findings by weighting results from individual studies and controlling for multiple moderators. This method has been successfully applied to the study of major depressive disorder (23), childhood maltreatment (31), and bipolar disorder (32). For significant findings, we then conducted subgroup meta-analysis on TBSS results and performed a meta-regression analysis to examine effects of demographic and clinical characteristics on the discovered white matter microstructural alterations. We hypothesized that decreased FA was manifest in TS patients when compared with HCs, especially in motor- or vocal-related tracts.

## Method

### Inclusion of Studies

We searched relevant studies from PubMed, Medline, and Web of Science published up to December 2020 with keywords “Tourette syndrome” or “Gilles de la Tourette syndrome” plus “diffusion tensor imaging” or “DTI” or “fractional anisotropy” or “FA” or “white matter.” We also manually checked the reference lists of the retrieved articles for additional relevant studies. All studies included in this meta-analysis are according to the Preferred Reporting Items for Systematic Reviews and Meta-Analyses (PRISMA) guidelines (33).

The inclusion criteria were as follows: (1) studies in which patients were diagnosed according to *Diagnostic and Statistical Manual of Mental Disorders, 4th Edition* (DSM-IV); (2) studies that compared whole-brain FA alteration between patients with TS and HCs; (3) studies that used the VBA or TBSS approach for DTI data analysis; (4) studies whose results were based on Montreal Neurological Institute (MNI) or Talairach coordinates; and (5) studies that were published in English in a peer-reviewed journal.

The exclusion criteria were as follows: (1) case reports or reviews; (2) studies with < 10 participants in either TS or HC group; and (3) peak coordinates could not be retrieved from the published article or after contacting the authors.

### Quality Assessment and Data Extraction

Two authors (C.M.Y. and L.Y.) independently searched the literature, assessed the quality of the retrieved articles, and extracted and cross-checked the data. In cases of disagreement, a third author helped to reach consensus. We assessed study quality using a 12-point checklist (see Supplementary Material) which was divided into three categories: participants (items 1–4), methods for image acquisition and analysis (items 5–10), and results and conclusions (items 11 and 12). Each item was given a score of 1, 0.5, or 0 to indicate whether the criteria were fully met, partially met, or unfulfilled, respectively, and any study scoring > 6.0 was included in the meta-analysis.

We extracted the following variables from each selected study: the characteristics of the participants and their illness (sample size, mean age of participants, sex, symptom severity, and drug status); magnetic resonance methodology (magnetic field strength, acquisition voxel size, number of diffusion directions, and type of analysis); statistical methodology (statistical threshold and correction methods for multiple comparisons); and 3D coordinates (for voxel-level quantitative meta-analyses).

### Voxel-Wise Meta-Analysis: AES-SDM

We analyzed FA differences in white matter between patients with TS and HCs following the standardized process of AES-SDM (www.sdmproject.com) (30) and the detail flow chart was showed in Supplementary Figure 1. Briefly, the AES-SDM technique uses effect size to combine reported peak coordinates that are extracted from statistical parametric maps, which recreates original maps of the effect size of FA difference in white matter between patients and controls. The created maps were visualized by MRIcron software (www.mricro.com/mricron/) overlaid on a high-resolution brain template generated by the International Consortium for Brain Mapping. To allow combination of VBA and TBSS studies, we adopted the TBSS template included in AES-SDM, which has been used and described by Wise and colleagues (32). All analytical processes followed the SDM tutorial (https://www.sdmproject.com/software/tutorial.pdf) and related publication (30). We adopted the default AES-SDM thresholds (anisotropy = 1.0; full width at half maximum = 20 mm, voxel *p* = 0.005, peak height *Z* = 1, cluster extent = 10 voxels) (34). The AES-SDM results are represented on a 3D-rendered brain, removing part of the left or right hemisphere and highlighting areas of the brain with significant FA alterations (35).

### Jackknife Sensitivity Analysis and Subgroup Analysis

To assess the robustness of the findings, we conducted a systematic whole-brain voxel-based jackknife analysis, in which we iteratively repeated the analysis, excluding one dataset at a time to establish the extent to which the results could be replicated. If a brain region remained significant in all or most of the combinations of studies, we considered the finding to be highly replicable (36). This was done both for combining studies with TBSS and VBA and for studies with TBSS alone. We did not perform meta-analysis in other subgroups (i.e., VBA method, adults, adolescents, medicated patients, and drug-free patients) due to the lack of sufficient samples to draw reliable conclusions.

### Analysis of Heterogeneity and Publication Bias

Heterogeneity refers to between-study variations. We conducted a between-study heterogeneity analysis of individual clusters using a random-effects model with Q statistics, with thresholds of *p* = 0.005, peak *Z* = 1.00, and a cluster extent of 10 voxels. Areas showing significant heterogeneity overlapping with the main findings were explored using meta-regression analyses to search for the sources of between-study variability. We also assessed publication bias by testing funnel plots *via* the Egger test in which any result showing *p* < 0.05 was regarded as having a significant publication bias.

### Meta-Regression Analysis

To characterize potential effects of key demographic and clinical variables on white matter, we performed meta-regression analysis using age, percentage of male patients, symptom severity [Yale Global Tic Severity Scale (YGTSS) (37)], and illness duration as independent variables in each study. The results were weighted by the square root of the sample sizes. To minimize spurious relationships, we selected a more conservative threshold of *p* = 0.0005 as used in previous studies (23, 30), requiring abnormalities to be detected both in the slope and in one of the extremes of the regressors, and discarded findings in regions other than those detected in the main analysis. We displayed the main output for each variable in a map of regression slope. Finally, we visually inspected regression plots to discard fittings that were obviously driven by too few studies (36).

### Fiber Tracking

The DTI query software allows placement and interactive manipulation of box-shaped region of interest (ROI) to display pathways passing through specific anatomic areas, making it easier to explore and interpret white matter pathways (38). We used this technique to display the most probable white matter tracts passing through clusters of voxels that showed significant FA group differences with an atlas of human white matter anatomy (39). The white matter tracts were mapped using streamline tracking techniques as well as filtered by tract length and ROI centered on the coordinates of significant clusters.

## Results

### Included Studies and Sample Characteristics

**Figure 1:**
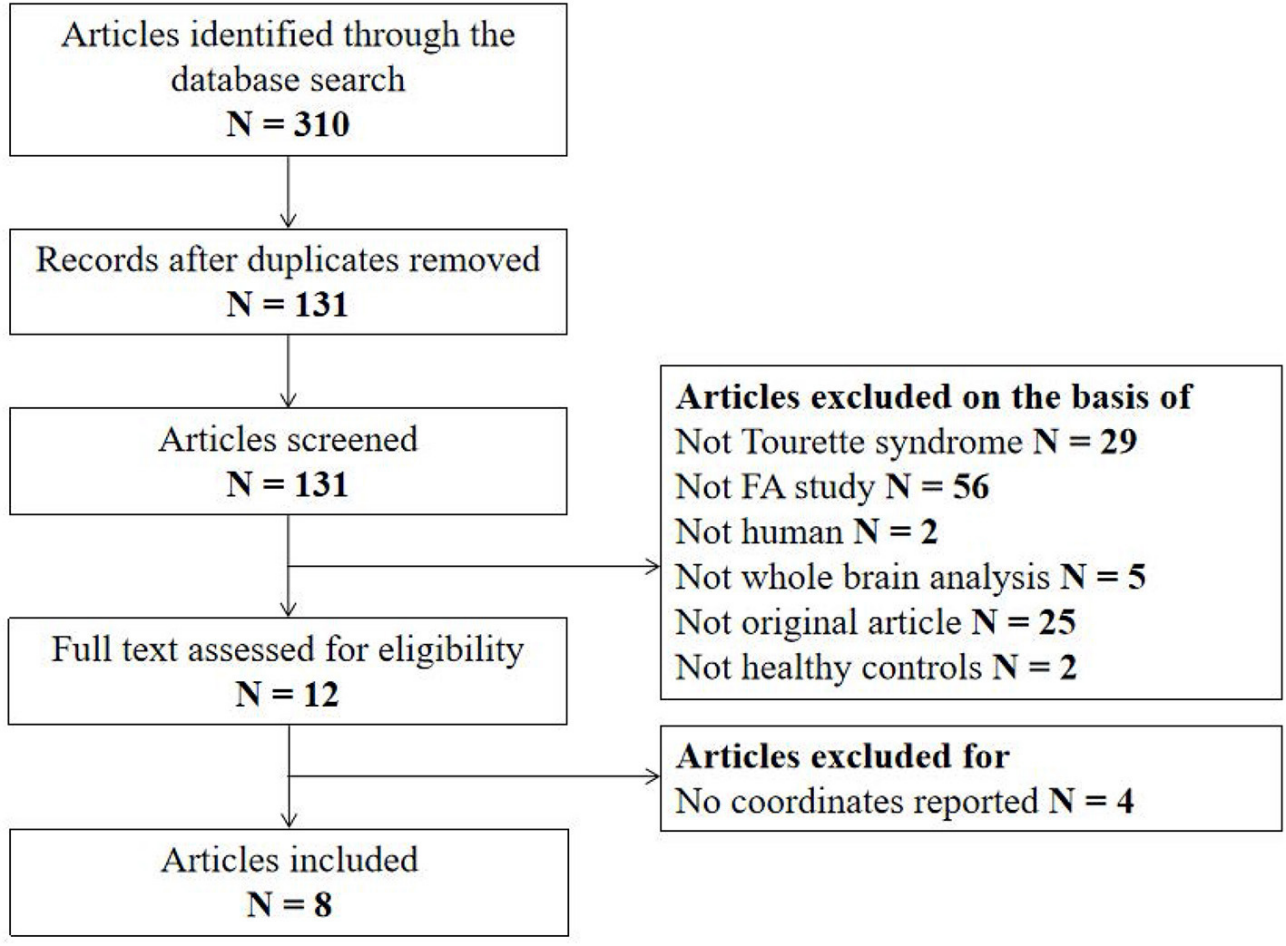
Flow diagram for the identification and exclusion of studies. N, number; FA, fractional anisotropy.

The search strategy identified 310 potentially relevant studies, eight of which met our inclusion criteria. The flow chart for study inclusion is shown in Figure 1. A total of 168 patients with TS (mean age 20.0 years) and 163 HCs (mean age 20.1 years) were included. The clinical and demographic data from all included studies are summarized in Table 1. Among eight studies, five recruited 106 patients who were either never medicated or without medication at the recruitment point. Due to high comorbidity rate, only 45 patients in three studies had one diagnosis, and the others were simultaneously diagnosed with ADHD, OCD, anxiety disorder, or depression. FA alterations involved a total of 33 coordinates in the included studies. The quality scores, ranging from 10.5 to 12 (mean score 11; Table 1; Supplementary Table 1), demonstrated that the included studies were of high quality, ensuring a more exhaustive and accurate meta-analysis. The technical details and main findings of all included studies are summarized in Table 2.

**Table 1:** Demographic and clinical characteristics of the participants in the eight studies included in the meta-analysis.

**References**	**No. (male)**		**Age [year (mean** **±** **SD)]**		**Illness duration [year (mean ± SD)]**	**Severity (YGTSS) (mean ± SD)**	**Statistical threshold**	**Medication %**	**Quality scores**
	**TS**	**HCs**	**TS**	**HCs**					
Sigurdsson et al. (27)	28 (25)	30 (28)	14.5 ± 3.8	14.1 ± 2.9	NA	33.0 ± 15.3	*p* < 0.05 (FWE)	29	11
Wen et al. (11)	27 (20)	27 (20)	9.0 ± 3.4	10.7 ± 3.3	1.8 ± 1.4	46.5 ± 18.0	*p* < 0.05 (TFCE)	Drug naïve	10.5
Müller-Vahl et al. (25)	19 (19)	20 (20)	30.4 ± 11.0	31.7 ± 10.9	NA	28.8	*p* < 0.001 (FWE)	Drug naïve/drug free	10.5
Jeppesen et al. (28)	24 (NA)	18 (NA)	NA	NA	4.6 ± 1.9	17.5 ± 11.1	*p* < 0.05 (TFCE)	Drug free	10
Liu et al. (29)	21 (20)	20 (17)	7.9 ± 2.0	8.1 ± 2.3	1.8 ± 0.6	41.7 ± 12.5	*p* < 0.05 (TFCE)	Drug naïve/drug free	12
Govindan et al. (17)	15 (12)	14 (6)	11.6 ± 2.5	12.3 ± 3.2	NA	13.7	*p* < 0.05 (MC)	67	11
Neuner et al. (24)	19 (13)	19 (12)	30.1 ± 10.8	28.9 ± 8.5	NA	52.0 ± 16.7	*p* < 0.05 (FWE)	58	11.5
Thomalla et al. (26)	15 (13)	15 (13)	34.5 ± 8.9	34.6 ± 9.1	26.5 ± 8.5	42.0 ± 16.0	*p* < 0.001 (FDR)	Drug naïve/drug free	11.5

*TS, Tourette syndrome; HCs, healthy controls; No., number; SD, standard deviation; NA, not applicable; YGTSS, Yale Global Tie Severity Scale; FWE, family-wise error; TFCE, threshold-free cluster enhancement; FDR, false-discovery rate; MC, multiple comparison*.

**Table 2:** Technical details and main findings of the eight studies included in this meta-analysis.

**References**	**Field**	**Acquisition voxel (mm^**3**^)**	**No. of directions**	**Coordinate system**	**No. of coordinates**	**Type of analysis**	**Main findings of FA**
Sigurdsson et al. (27)	3 T	1 × 1 × 2	32	MNI	0	TBSS	Negative
Wen et al. (11)	3 T	1 × 1 × 1	30	MNI	12	TBSS	Decreased FA in the body of corpus callosum, forceps major, right inferior longitudinal fasciculus, right inferior fronto-occipital fasciculus, right corticospinal tract, left frontal lobe subgyral, left anterior thalamic radiations, and bilateral superior longitudinal fasciculus
Müller-Vahl et al. (25)	1.5 T	NA	12	MNI	11	VBA	Decreased FA in the bilaterally in the medial frontal gyrus, the pars opercularis of the left inferior frontal gyrus, the middle occipital gyrus, the right cingulate gyrus, and the medial premotor cortex
Jeppesen et al. (28)	3 T	1 × 1 × 1	15	MNI	0	TBSS	Negative
Liu et al. (29)	1.5 T	NA	15	MNI	0	TBSS	Negative
Govindan et al. (17)	3.0 T	3 × 3 × 3	6	MNI	0	TBSS	Negative
Neuner et al. (24)	1.5 T	2 × 2 × 2	30	MNI	9	TBSS	Decreased FA in the corticospinal tract, the corpus callosum and long association fiber pathways such as the inferior fronto-occipital fascicle and the superior longitudinal fascicle as well as in the uncinate fascicle
Thomalla et al. (26)	3 T	2 × 2 × 2	24	MNI	1	VBA	Increased FA in the mesial part of the left central and postcentral gyrus

*No., number; FA, fractional anisotropy; MNI, Montreal Neurological Institute; TBSS, tract-based spatial statistics; VBA, voxel-based analysis*.

### Pooled Meta-Analysis of All Included Studies

**Figure 2:**
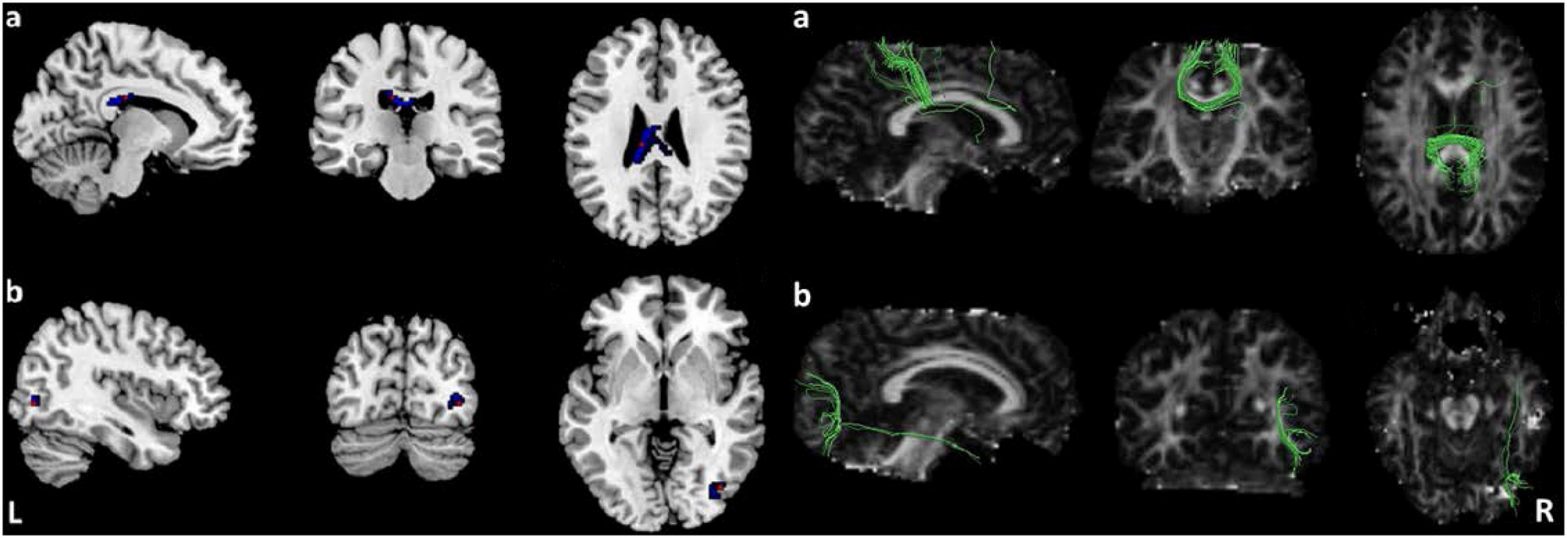
Results of pooled meta-analysis. Decreased FA of the CC **(a)** and right ILF **(b)** in TS patients compared with HCs and cluster-related white mater diffusion tensor tracts. Regions with blue color area showed lower FA in TS, red dots represent the peak coordinate. FA, fractional anisotropy; TS, Tourette syndrome; HCs, healthy controls; CC, corpus callosum; ILF, inferior longitudinal fasciculus.

Pooled meta-analysis revealed two regions of decreased FA in TS patients compared with HCs: the white matter of the CC [MNI −10/−24/26, splenium of CC (SCC) *Z* = −1.573, *p* < 0.001] and right inferior longitudinal fasciculus (ILF, MNI 40/−76/−2, *Z* = −1.594, *p* < 0.001) (Table 3; Figure 2). White matter tracts traversing through these voxels were displayed in a bounding box size of 6.0 × 6.0 × 6.0 mm^3^ with DTI query software (Figure 2). No significant FA increase was found in patients with TS compared with HCs.

**Table 3:** Clusters of fractional anisotropy reductions in all patients with TS relative to HCs.

**Region**	**MNI coordinates**	**SDM**	***p*-value**	**No. voxels**	**Cluster breakdown**
	***x*, *y*, *z***	***Z*-value**	**uncorrected**		**(voxels)**
Corpus callosum	−10, −24, 26	−1.573	<0.001	114	Corpus callosum (114)
Right inferior network, inferior longitudinal fasciculus	40, −76, −2	−1.594	<0.001	27	Right inferior longitudinal fasciculus (19); right middle occipital gyrus, BA 19 (6); right inferior occipital gyrus, BA 19 (1) (undefined), BA 19 (1)

*TS, Tourette syndrome; HCs, healthy controls; MNI, Montreal Neurological Institute; SDM, signed differential mapping; No., number; BA, Brodmann area*.

### Subgroup Meta-Analysis

The subgroup meta-analysis of TBSS method included six datasets that compared 134 patients with TS to 128 HCs. Decreased FA in the CC was significant in the subgroup analysis (MNI −10/−22/26, *Z* = −1.605, *p* < 0.001), while changes of the right ILF was not found (Table 4).

**Table 4:** Clusters of fractional anisotropy reductions in TBSS method studies.

**Region**	**MNI coordinates**	**SDM**	***p*-value**	**No. voxels**	**Cluster breakdown**
	***x*, *y*, *z***	***Z*-value**	**uncorrected**		**(voxels)**
Corpus callosum	−10, −22, 26	−1.605	<0.001	143	Corpus callosum (142)
					Left median network, cingulum (1)

*TBSS, tract-based spatial statistics; MNI, Montreal Neurological Institute; SDM, signed differential mapping; No., number*.

### Jackknife, Heterogeneity, and Publication Bias Analysis

Whole-brain jackknife sensitivity analysis showed that decreased FA in CC in patients was highly replicable, being preserved in all but one combination (24); decreased FA in the right ILF remained significant in all but two combinations (11, 25) (Table 5). In the TBSS subgroup, the FA reduction in the CC was preserved in all combinations (Supplementary Table 2).

**Table 5:** Results of jackknife analysis in all included studies.

**Discarded study**	**Decreased FA**	
	**Corpus callosum**	**Right inferior network, inferior longitudinal fasciculus**
Govindan et al. (17)	Yes	Yes
Müller-Vahl et al. (25)	Yes	No
Jeppesen et al. (28)	Yes	Yes
Liu et al. (29)	Yes	Yes
Neuner et al. (24)	No	Yes
Sigurdsson et al. (27)	Yes	Yes
Thomalla et al. (26)	Yes	Yes
Wen et al. (11)	Yes	No
Total	7/8	6/8

*FA, fractional anisotropy*.

**Figure 3:**
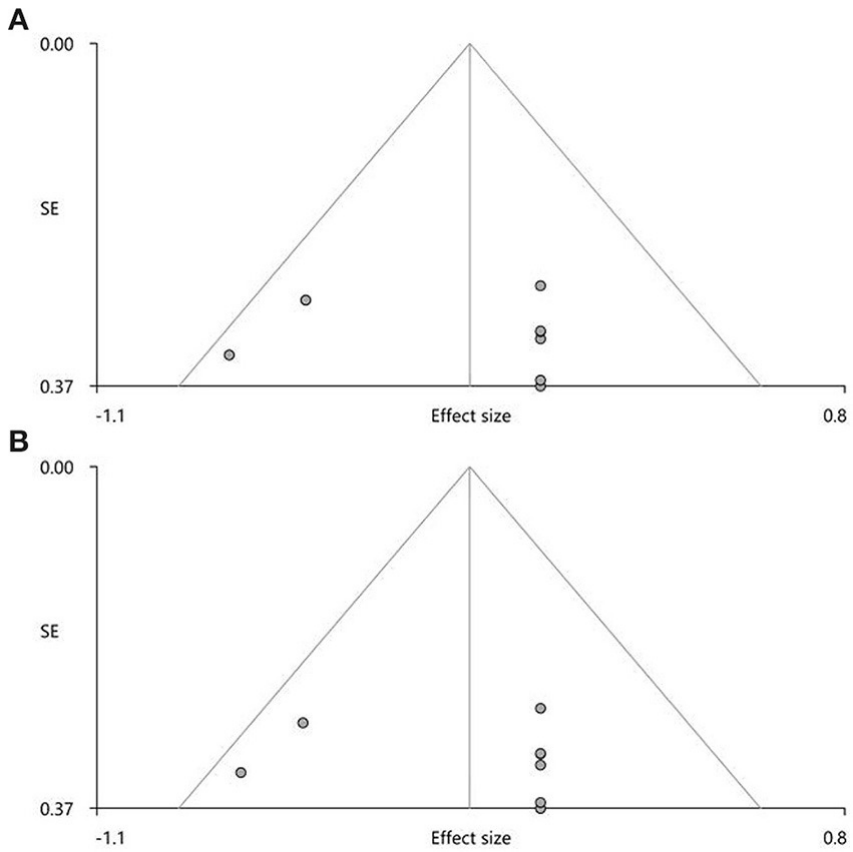
Results of funnel plot analysis to test for publication bias. For the pooled meta-analysis, the Egger's test and funnel plots revealed no significant publication bias **(A)** in the corpus callosum (*Z* = 0.75, *t* = 0.22, *df* = 6, *p* = 0.832) and **(B)** in the right inferior network, inferior longitudinal fasciculus (*Z* = 1.14, *t* = 0.34, *df* = 6, *p* = 0.747).

In the pooled meta-analysis, none of the regions with altered FA (Table 3) showed statistically significant heterogeneity between studies. Funnel plots demonstrated that the main findings were driven by at least six studies (Figure 3). Analysis of publication bias showed that the Egger test was non-significant for the CC (*p* = 0.832) and right ILF (*p* = 0.747).

### Meta-Regression Analysis

Age, sex, tic symptom severity (YGTSS), and illness duration were not significantly associated with TS-related white matter FA changes from the meta-regression analysis.

## Discussion

To our knowledge, this is the first quantitative meta-analysis integrating DTI studies in patients with TS. Voxel-wise meta-analysis using AES-SDM in the present study supported that patients were associated with decreased FA in CC and right ILF. These results were robust under jackknife analysis. Clinical parameters including age, sex, tic severity, and illness duration, did not show significant association with white matter microstructural alterations.

The decreased FA in CC (mainly SCC) was found in TS patients compared with HCs in the present study, which is consistent with numerous previous studies (9–11). For example, it has previously been shown that FA values in the posterior portions of the CC (including SCC) were significantly lower in monozygotic twins of TS patients (9). Another structural study for deep white matter tracts revealed decreased FA in right SCC, and greater alterations were correlated with more severe tic symptoms in patients (11). The CC is the largest fiber bundle of the human brain connecting the left and right cerebral hemispheres while the SCC predominantly connects bilateral parietal and temporal areas (40). In line with our findings, Worbe et al. (14) suggested that patients with complex tics were associated with cortical thinning in parietal regions by showing that cortical thinning in parietal and temporal cortices was correlated negatively with the tic severity. Moreover, temporo-parietal junction (TPJ) is a functionally defined region pivotal to social cognition (41), and the dysfunction of TPJ could bring negative influence on conscious human experience and impact mental health (42). Task-fMRI studies (12, 13) on mental state judgments have demonstrated abnormal task-related activity in the right TPJ in patients, which was further shown to be related to the tic symptom. Therefore, the FA reduction in the SCC in the current study suggested impaired parietal and temporal interhemispheric connectivity, which might lead to neurocognitive deficits in mental and physical aspects of individuals with TS.

As the primary inhibitory neurotransmitter in the brain, g-aminobutyric acid (GABA) has been increasingly recognized to be involved in the pathophysiology of TS (43). Current evidence suggested that the tic symptom of TS was associated with dysfunction of GABA (44, 45), while GABAergic drugs have been shown to improve tics in a safe and effective way (46, 47). With the measurement of *in vivo* brain GABA non-invasively, a magnetic resonance spectroscopy (MRS) study demonstrated that GABA concentrations within the supplementary motor area (SMA) was linked to the pathogenesis of tics in TS and correlated with FA values within the CC (48). Furthermore, GABA concentrations within the SMA could be predicted by tic severity and FA values in the CC. Based on these findings, we suggested that decreased FA in the CC observed in our meta-analysis might provide new insight into treatment target in patients with TS. Future studies are needed to articulate the specific role of CC in GABA-related mechanisms underlying TS.

The present meta-analysis also identified significantly decreased FA in the right ILF in patients with TS compared with HCs. Previous DTI study combined both TBSS and atlas-based ROI analysis demonstrated a reduction of FA in the right ILF in TS patients, which was negatively associated with tic severity (11). The ILF is widely accepted as a direct connection between the occipital cortex and the temporal lobe (49) and is functionally correlated with thought disorders, visual emotion, and cognitive impairments (50). In addition, Latini and colleagues demonstrated that the ILF played an important role in integrating information of visual, memory, and emotions (51), where deficits of visual memory in TS were found to be related to occipital dysfunction (15), and cognitive disturbances were correlated with abnormal brain activity in temporal areas (16). The peak of altered FA located in the right middle and inferior occipital gyrus. There were several studies that provided evidence of gray matter alteration in the occipital brain region in patients with TS, including changed cortical thickness, cortical sulcus, and cortical curvature in occipital gyrus (52), and the abnormal functional connectivity was also found in the occipital area in patients with TS (53). In sum, our finding of the decreased FA in ILF (mainly in the occipital part) might underlie the deficits in visual emotional modulation and cognitive processing in patients with TS.

We conducted subgroup analysis confined to the TBSS method. Compared with the pooled meta-analysis, the subgroup revealed FA reduction only in the CC with larger clusters, but not in the right ILF. This finding was consistent with the previous study showing that TBSS may detect white matter abnormalities more accurately than VBA (32). The VBA method was not conducted in the subgroup analysis because there were only two original studies. Nevertheless, it was still worth noting that one of the VBA study also showed abnormal FA involving the posterior part of the CC (26). Hence, the FA reduction in the CC might be regarded as a stable indicator in TS and the TBSS technique was recommended to explored white matter microstructural abnormalities in this disorder.

The meta-regression analysis revealed that there were no significant associations between white matter microstructural alterations and examined demographic and clinical variables. Consistent with our findings, Plessen et al. have investigated the small size (on the midsagittal slice) and reduced FA values of CC in children with TS, respectively (10, 54). The results were not influenced by medication or comorbid illnesses. Furthermore, there were no significant correlations between reduction of FA in TS and the severities of tic symptoms (10). However, some studies provided evidence for a close relationship between the white matter microstructure of CC and tic severity (25, 55). Considering the small sample size for the included studies and potential confounding factors, the meta-regression analysis may result in negative results and future studies are needed for replication.

## Limitation

This study has some limitations. Firstly, like most voxel-based meta-analyses, our study was based on summarized data (i.e., coordinates and effect sizes from published studies) rather than raw image files from original data (34), which may limit the precision of the spatial location of the observed effects. Second due to the high comorbidity rate of the included samples, the influences of medication treatment and comorbidities (e.g., ADHD or/and OCD) cannot be removed. As a result, future meta-analysis research is warranted when sufficient data on medication-naive patients without comorbidities are available in the literature. Thirdly, subgroup analysis was not able to be conducted comparing adults vs. adolescents and medicated patients vs. drug-free subjects, and therefore specific effects of age and medication status on the white matter microstructure need to be investigated in the future.

## Conclusion

The present meta-analysis of DTI studies identified robust white matter deficits in the CC and right ILF in patients with TS, providing a strong evidence for disturbances in the interhemispheric connections and long association fiber bundles in the right hemisphere in TS. Because of limited studies in the literature, future investigations with larger sample size are warranted to provide further evidence for the white matter changes in TS.

## Data Availability Statement

The original contributions presented in the study are included in the article/Supplementary Material, further inquiries can be directed to the corresponding author/s.

## Author Contributions

SL contributed to the conception and design of the study, as well as the supervision of all the work of this review. CY and LY contributed to literature searching and drafting of the manuscript. All authors made critical revision of the manuscript for important intellectual content and gave final approval of the version to be submitted.

## Funding

This study was supported by the National Natural Science Foundation of China (Grant Nos. 82120108014, 82071908, 81621003, 81671664, and 82102007), 1.3.5 Project for Disciplines of Excellence, West China Hospital, Sichuan University (Project Nos. ZYYC08001 and ZYJC18020), Sichuan Science and Technology Program (Grant Nos. 2021JDTD0002, 2020YJ0018, and 2021YFS0077), and Science and Technology Project of the Health Planning Committee of Sichuan (Project Nos. 19PJ078 and 20PJ010).

## Conflict of Interest

WZ consulted to VeraSci. The remaining authors declare that the research was conducted in the absence of any commercial or financial relationships that could be construed as a potential conflict of interest.

## Publisher's Note

All claims expressed in this article are solely those of the authors and do not necessarily represent those of their affiliated organizations, or those of the publisher, the editors and the reviewers. Any product that may be evaluated in this article, or claim that may be made by its manufacturer, is not guaranteed or endorsed by the publisher.
