# Supplementary material for: Microstructural Abnormalities of White Matter Across Tourette Syndrome: A Voxel-Based Meta-Analysis of Fractional Anisotropy
Source: Front Neurol. 2021 Sep 9;12:659250. doi: 10.3389/fneur.2021.659250 (PMC8458640; doi:10.3389/fneur.2021.659250)
Supplement: Supplementary file 1 [file Data_Sheet_1.docx]

Supplementary Material

Table S1: Imaging Methodology Quality Assessment Checklist (Compiled from 1-3)

| 12-points checklist | Govindan *et al.* (1) | Müller-Vahl *et* *al.* (2) | Liu *et al.* (3) | | Neuner *et al.* (4) | Sigurdsson *et al.* (5) | Thomalla *et al.* (6) | Wen *et al.* (7) | Jeppesen *et al.* (8) |
| --- | --- | --- | --- | --- | --- | --- | --- | --- | --- |
| Category 1: Subjects |  |  | |  |  |  |  |  |  |
| 1. Patients were evaluated prospectively, specific diagnostic criteria were applied, and demographic data was reported | 1 | 0.5 | | 1 | 1 | 1 | 1 | 1 | 0.5 |
| 2. Healthy comparison subjects were evaluated prospectively, psychiatric and medical illnesses were excluded, and demographic data was reported | 1 | 0.5 | | 1 | 0.5 | 0.5 | 0.5 | 0.5 | 0.5 |
| 3. Important variables (e.g. sample size, mean age of participants, sex, symptom severity, drug status, and comorbidities) were checked either by stratification or statistically | 1 | 1 | | 1 | 1 | 1 | 1 | 1 | 0.5 |
| 4. Sample size per group > 10, and no significant difference in age and sex existed | 1 | 1 | | 1 | 1 | 1 | 1 | 1 | 1 |
| Category 2: Methods for image acquisition and analysis |  |  | |  |  |  |  |  |  |
| 5. Magnet strength at least 1.5T | 1 | 1 | | 1 | 1 | 1 | 1 | 1 | 1 |
| 6. DTI with at least 12 directions was used to investigated fractional anisotropy differences | 0.5 | 1 | | 1 | 1 | 1 | 1 | 1 | 1 |
| 7. Whole brain analysis was automated with no a-priori regional selection | 1 | 1 | | 1 | 1 | 1 | 1 | 1 | 1 |
| 8. Coordinates reported in a standard space | 1 | 1 | | 1 | 1 | 1 | 1 | 1 | 1 |
| 9. The imaging technique used was clearly described so as it could be reproduced | 1 | 1 | | 1 | 1 | 1 | 1 | 1 | 1 |
| 10. Measurements were clearly described so that they could be reproduced | 1 | 0.5 | | 1 | 1 | 1 | 1 | 0.5 | 1 |
| Category 3: Results and conclusions |  |  | |  |  |  |  |  |  |
| 11. Statistical parameters for significant and important non-significant differences were provided | 1 | 1 | | 1 | 1 | 1 | 1 | 1 | 1 |
| 12. Conclusions were consistent with the results obtained and the limitations were discussed | 0.5 | 1 | | 1 | 1 | 0.5 | 1 | 0.5 | 0.5 |
| total scores | 11 | 10.5 | | 12 | 11.5 | 11 | 11.5 | 10.5 | 10 |

Table S2: Results of jackknife analysis in TBSS studies (6 studies).

| Discarded study | Decreased FA |
| --- | --- |
|  | Corpus callosum |
| Govindan *et al.* (1) | Yes |
| Liu *et al.* (3) | Yes |
| Jeppesen *et al.* (8) | Yes |
| Neuner *et al.* (4) | Yes |
| Sigurdsson *et al.* (5) | Yes |
| Wen *et al.* (7) | Yes |
| Total | 6/6 |

TBSS, tract-based spatial statistics; FA, fractional anisotropy.

Fig S1: The flow chart of data processing and analysis by AES-SDM.

Abbreviation: AES-SDM, anisotropic effect size-signed differential mapping

**Reference:**

1. Govindan RM, Makki MI, Wilson BJ, Behen ME, Chugani HT. Abnormal Water Diffusivity in Corticostriatal Projections in Children with Tourette Syndrome. Hum Brain Mapp. 2010;31:1665-1674.

2. Müller-Vahl KR, Grosskreutz J, Prell T, Kaufmann J, Bodammer N, Peschel T. Tics Are Caused by Alterations in Prefrontal Areas, Thalamus and Putamen, While Changes in the Cingulate Gyrus Reflect Secondary Compensatory Mechanisms. Bmc Neurosci. 2014;15:6.

3. Liu Y, Miao W, Wang J, Gao P, Yin G, Zhang L, et al. Structural Abnormalities in Early Tourette Syndrome Children: A Combined Voxel-Based Morphometry and Tract-Based Spatial Statistics Study. Plos One. 2013;8:e76105.

4. Neuner I, Kupriyanova Y, Stocker T, Huang R, Posnansky O, Schneider F, et al. White-Matter Abnormalities in Tourette Syndrome Extend Beyond Motor Pathways. Neuroimage. 2010;51:1184-1193.

5. Sigurdsson HP, Pepes SE, Jackson GM, Draper A, Morgan PS, Jackson SR. Alterations in the Microstructure of White Matter in Children and Adolescents with Tourette Syndrome Measured Using Tract-Based Spatial Statistics and Probabilistic Tractography. Cortex. 2018;104:75-89.

6. Thomalla G, Siebner HR, Jonas M, Baumer T, Biermann-Ruben K, Hummel F, et al. Structural Changes in the Somatosensory System Correlate with Tic Severity in Gilles De La Tourette Syndrome. Brain. 2009;132:765-777.

7. Wen H, Liu Y, Wang J, Rekik I, Zhang J, Zhang Y, et al. Combining Tract- and Atlas-Based Analysis Reveals Microstructural Abnormalities in Early Tourette Syndrome Children. Hum Brain Mapp. 2016;37:1903-1919.

8. Jeppesen SS, Debes NM, Simonsen HJ, Rostrup E, Larsson HB, Skov L. Study of Medication-Free Children with Tourette Syndrome Do Not Show Imaging Abnormalities. Movement disorders : official journal of the Movement Disorder Society. 2014;29:1212-1216.
