# Supplementary figures and images for: Microstructural Abnormalities of White Matter Across Tourette Syndrome: A Voxel-Based Meta-Analysis of Fractional Anisotropy
Source: Front Neurol. 2021 Sep 9;12:659250. doi: 10.3389/fneur.2021.659250 (PMC8458640; doi:10.3389/fneur.2021.659250)

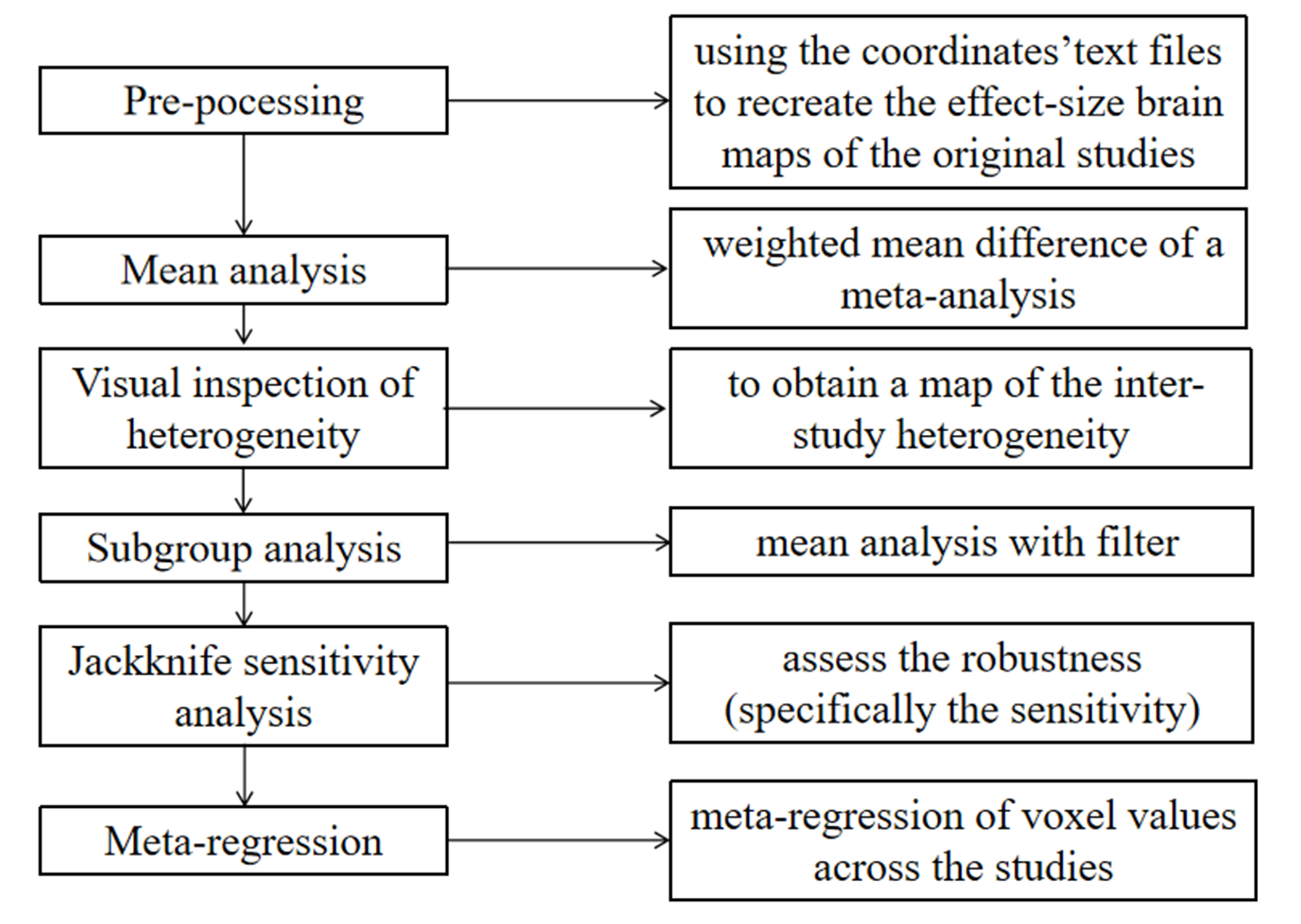

Supplement: Supplementary file 2 [file Image_1.tif]
